# Exome Sequence Analysis to Characterize Undiagnosed Family Segregating Motor Impairment and Dystonia

**DOI:** 10.3390/jcm13144252

**Published:** 2024-07-21

**Authors:** Ahmad M. Almatrafi, Abdulfatah M. Alayoubi, Majed Alluqmani, Jamil A. Hashmi, Sulman Basit

**Affiliations:** 1 Department of Biology, College of Science, Taibah University, Medina 42353, Saudi Arabia; 2 Center for Genetics and Inherited Diseases, Taibah University, Madinah 42353, Saudi Arabia; hashmi.ja@gmail.com; 3 Department of Basic Medical Sciences, College of Medicine, Taibah University, Madinah 42353, Saudi Arabia; aayoubi@taibahu.edu.sa; 4 Department of Neurology, College of Medicine, Taibah University, Medina 42353, Saudi Arabia; mloqmani@hotmail.com

**Keywords:** diagnosing undiagnosed, *SLC30A10*, pathogenic variants, neurodevelopmental disorders

## Abstract

**Background**: Hypermanganesemia with dystonia 1 (HMNDYT1) is a rare genetic disorder characterized by elevated blood manganese levels. This condition is associated with polycythemia, motor neurodegeneration with extrapyramidal features, and hepatic dysfunction, which can progress to cirrhosis in some patients. **Materials and Methods**: In this study, a consanguineous Saudi family with two affected individuals exhibiting symptoms of severe motor impairment, spastic paraparesis, postural instability, and dystonia was studied. Clinical and radiographic evaluations were conducted on the affected individuals. Whole exome sequencing (WES) was performed to diagnose the disease and to determine the causative variant underlying the phenotype. Moreover, Sanger sequencing was used for validation and segregation analysis of the identified variant. Bioinformatics tools were utilized to predict the pathogenicity of candidate variants based on ACMG criteria. **Results**: Exome sequencing detected a recurrent homozygous missense variant (c.266T>C; p.L89P) in exon 1 of the *SLC30A10* gene. Sanger sequencing was employed to validate the segregation of the discovered variant in all available family members. Bioinformatics tools predicted that the variant is potentially pathogenic. Moreover, conservation analysis showed that the variant is highly conserved in vertebrates. **Conclusions**: This study shows that exome sequencing is instrumental in diagnosing undiagnosed neurodevelopmental disorders. Moreover, this study expands the mutation spectrum of *SLC30A10* in distinct populations.

## 1. Introduction

Manganese (Mn) is an essential heavy metal that exhibits antioxidant activity and plays a vital role in multiple cellular metabolic pathways [1,2]. It acts as a cofactor for several enzymes involved in a variety of activities including immunity, blood clotting, growth of bone and connective tissues as well as neurotransmitter metabolism [2]. The level of Mn within the body is maintained through a tight homeostatic mechanism involving intestinal absorption and excretion by the liver via the biliary system [2,3]. In humans, the primary source of manganese is dietary intake of food rich with Mn such as whole grains, rice, nuts, leafy green vegetables, and seafood [4]. However, chronic manganese intoxication has been reported among workers in certain industries, such as welding, smelting, and mining [5,6]. In addition, Mn intoxication has been observed among drug addicts of methcathinone and patients who underwent parenteral nutrition [7].

Mn competes with other ions to bind to proteins, and when present in excessive levels, it can lead to a neurotoxic syndrome known as “manganism”. In the case of manganism, the metal tends to accumulate in the muscles, liver, and brain; specifically, in the basal ganglia (BG) [6,8]. Hypermanganesemia can result in damage to the structures within the basal ganglia, particularly the globus pallidus external segment (GPe) [9]. Individuals with manganism experience early behavioral disturbances, also known as “manganese madness”, which are followed by the emergence of a prominent extrapyramidal syndrome [10]. This syndrome shares similarities with dystonia (MIM 128100) and Parkinson’s disease (MIM 168600) and is commonly referred to as manganese-induced Parkinsonism [11,12].

Several studies have reported hypermanganesemia among children without a history of significant exposure to Mn from their environment [13,14,15,16]. These patients not only exhibit high levels of manganese in their blood but also display varying degrees of hepatic cirrhosis, polycythemia, and dystonia [14,15]. Tuschl et al. reported that the first two siblings from a consanguineous marriage suffered hypermagnesemia, polycythemia, and liver dysfunction, with the association of an extrapyramidal motor disorder, and suggested an autosomal recessive inherited pattern of the disease, which was later named hypermanganesemia with dystonia 1 (HMNDYT1, MIM 613280) [17]. In 2012, autosomal recessive mutations in the *SLC30A10* gene were reported to be associated with HMNDYT1 [12,13]. This discovery provided a significant opportunity to gain insights into the molecular mechanisms involved in Mn homeostasis. 

The solute carrier family 30-member 10 (*SLC30A10*) gene encodes a protein that is required for the transport of manganese (Mn) from the cytosol to an extracellular pool [12]. The human *SLC30A10* is located on chromosome 1q41 and it consists of four coding exons [13]. In the current study, we reported a family with two affected individuals segregating HMNDYT1 in an autosomal recessive manner. Clinical and genetic investigation was performed and a homozygous missense variant (c.266T>C; p.L89P) was identified in the first exon of the *SLC30A10* gene. This mutation has been demonstrated previously to cause a complete loss of function of the SLC30A10 protein resulting in manganese neurotoxicity.

## 2. Materials and Methods

### 2.1. Patient Recruitment and Ethical Approval

**Figure 1:**
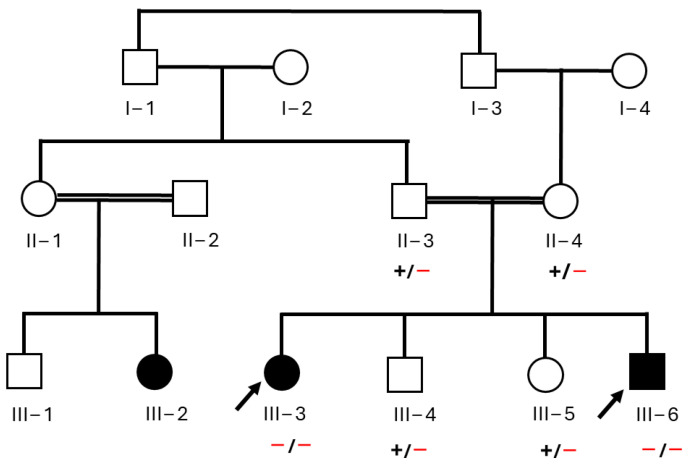
Pedigree of a Saudi family recruited for this research. Black arrows indicate the two affected individuals who clinically and genetically investigated in the study.

A Saudi family with two affected children (Figure 1), displaying symptoms of severe motor impairment, spastic paraparesis, postural instability, and dystonia, was recruited from King Fahad Hospital (KFH) in Madinah, Saudi Arabia. The initial screening of both patients was performed by a consultant neurologist at KFH. A blood test was performed and radiographs including X-rays, magnetic resonance imaging (MRI), and computed tomography scan (CT scan) were taken. Due to the inconclusive diagnosis of the disease, the family was referred for genetic testing at the Center of Genetic and Inherited Disease in Madinah to identify the underlying cause. Parents were interviewed regarding family medical history, and a pedigree chart was constructed (Figure 1). The study purpose was explained in detail to the parents, who provided written informed consent. The study received approval from Taibah University institutional review board (TUCDREC/27032021). Blood samples were collected from all family members, including both parents (II-3 and II-4), the two affected children (III-3 and III-6), and two additional unaffected siblings (III-4 and III-5). Samples were collected in ethylenediaminetetraacetic acid tubes for exome sequencing. 

### 2.2. Genomic DNA Isolation and Exome Sequencing

Genomic DNA was isolated from blood samples using the DNeasy Blood & Tissue Kit (Qiagen cat no. 69504) following the manufacturer’s instructions. DNA purity was assessed through 1.5% of agarose gel electrophoresis. DNA concentration was measured using a nanodrop spectrophotometer. DNA from both affected individuals including a female (III-3) and a male (III-6) was subjected to exome sequencing using the Illumina NovaSeq6000 according to a previously described protocol [18,19]. The xGen Exome Research Panel v2 kit (Integrated DNA Technologies cat no. 10005151) was used for DNA library preparation. The analysis of raw reads was performed using the Burrows–Wheeler Aligner (BWA) software (Version 0.7.17). The Illumina BaseSpace analysis tool was used for variant calling and filtration. The called variants were annotated using ANNOVAR software [https://annovar.openbioinformatics.org/en/latest/; accessed on 5 March 2024].

### 2.3. Exome Data Analysis and Sanger Sequencing 

Due to consanguineous marriage and clear autosomal recessive inheritance pattern of the phenotype, an analysis of exome data was carried out with a focus on homozygous and compound heterozygotes variants. Candidate variant selection, filtration, and prioritization were carried out based on ACMG guidelines. To predict the pathogenicity of the identified variants, various bioinformatics tools were employed including VarSome [https://varsome.com/; accessed on 3 April 2024], CADD [https://cadd.gs.washington.edu//; accessed on 3 April 2024], Mutation Assessor [http://mutationassessor.org/; accessed on 3 April 2024], MutationTaster [https://www.mutationtaster.org/; accessed on 4 April 2024], and PolyPhen-2 [https://genetics.bwh.harvard.edu/pph2/; accessed on 4 April 2024]. The candidate variants’ frequencies among the general population were inspected using version 4.1 of The Genome Aggregation Database (gnomAD) [https://gnomad.broadinstitute.org/; accessed on 4 April 2024], The International Genome Sample Resource (1000 genomes) [https://www.internationalgenome.org/; accessed on 4 April 2024], and in-house and exome data of 125 healthy Saudi individuals.

Bidirectional Sanger sequencing was conducted to analyze all potential variants detected in the samples of affected individuals (III-3 and III-6) and across all family members according to the earlier established protocol [18,20]. Specific primers were designed for each candidate variant using Primer3 software (version 0.4.0). A reference DNA sequence of candidate genes was obtained from the Ensembl genome browser [https://www.ensembl.org; accessed on 6 April 2024]. The Sanger sequence data were aligned with the target reference sequence using BIOEDIT software (version 6.0.7) to validate the identified variants.

### 2.4. Protein–Protein Interaction

NCBI protein (http://www.ncbi.nlm.nih.gov/protein/; accessed on 15 April 2024) was used to determine the amino acid sequence conservation of SLC30A10 across various organisms. The UNIPROT database (https://www.uniprot.org accessed on 15 April 2024) was used to map the identified pathogenic variant to the specific SLC30A10 protein domain. Additionally, the STRING website (version 12) was employed to explore the protein interaction network of SLC30A10. 

## 3. Results

### 3.1. Clinical Features of Affected Individuals

The family involved in the current study consists of six members of Arab ethnicity. The eldest affected individual (III-3) was a 12-year-old female at the time of enrolment and was the oldest offspring of the family. The affected individual (III-6) was a 2 year-old male and was the youngest family member. Both patients were born following uneventful pregnancies with immediate cry after normal vaginal delivery. They developed normally during their first year of life without any significant medical issues, infection, or trauma and never underwent surgery. They reached typical developmental milestones, including walking, communicating, and speaking normally. 

However, when patient III-3 reached the age of 2 years, her health started to deteriorate. She displayed behavioral changes such as extensive crying and isolation. Subsequently, she experienced a regression in motor development, leading to an inability to walk independently and difficulties with eating and swallowing. After a few years, she began to experience focal seizures, her condition worsened, and she became wheelchair bound.

The youngest affected individual, III-6, initially appeared to be fine until the age of one year. However, he later started to exhibit symptoms similar to those of patient (III-3), which gradually progressed over time. Currently, he is bedridden due to the severity of his symptoms.

During their medical follow-up, both affected individuals exhibited symptoms including dystonia, limb hypertonia, severe fine motor impairment, bradykinesia, rigidity, postural instability, and spastic paraparesis (Table 1). However, no signs of tremor were observed in either patient. In recent months, patients III-3 started experiencing hematemesis (vomiting blood). A diagnosis of liver cirrhosis was made, and unfortunately, she died because of complications of cirrhosis. The healthy individuals (II-3, II-4, III-4, and III-5) did not present any abnormal clinical phenotypes.

### 3.2. Brain MRI Finding

**Figure 2:**
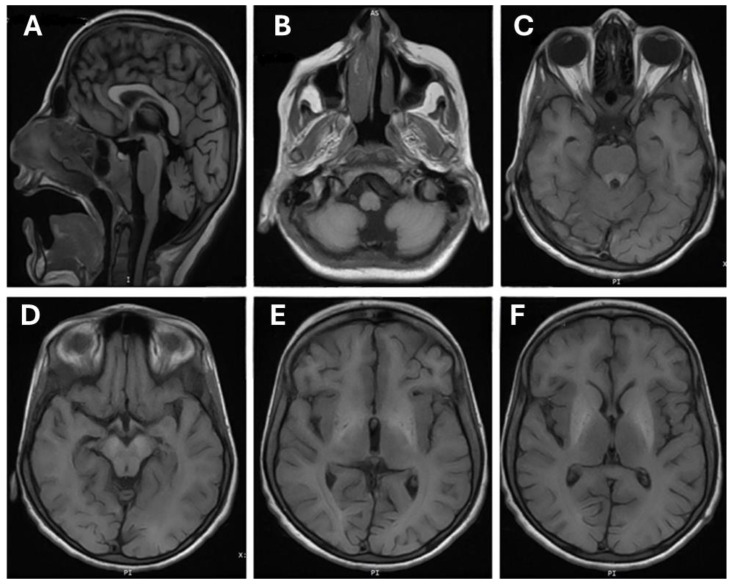
Brain magnetic resonance imaging (MRI) of patient (III-3). (**A**) Cerebellar atrophy flair saggital sequence flair; (**B**) hypoplasia of vermis axial T1; (**C**) increased signal from the basal ganglia down to the brain stem mainly midbrain (cerebri) and pones (ventral). Ventrical pons around 4th ventricle; (**D**) midbrain (cerebri bilateral); (**E**) frontal bilateral white matter hypersignal; (**F**) continuation of hypersignal to basal ganglia signs of hypomyelination.

A brain MRI performed on individual (III-3) at the age of 11 years suggests evidence of bilateral symmetrical signal alteration involving the basal ganglia, particularly the globus pallidi and substantia nigra, with suspicious involvement of the dorsal brainstem and dentate nuclei (Figure 2). These alterations appear as high signal intensity on T1-weighted images (T1-WI), with no observed signal alteration on T2-WI. A blooming effect is seen only in the globus pallidi, indicating the iron/or manganese deposition. However, no diffusion restriction is noted.

The differentiation between grey and white matter is preserved. Moreover, there are no signs of acute intracranial infarction or hemorrhage. Furthermore, the ventricular system and subarachnoid spaces appear normal, without midline shift, hydrocephalus, brain edema, or herniation. The cerebellum shows diffuse mild atrophy. The imaged vessels are patent, and the orbits and mastoid air cells show no abnormalities. Chronic inflammatory changes are observed in the sinuses. 

### 3.3. Exome Data Analysis and Genetic Assessment 

**Figure 3:**
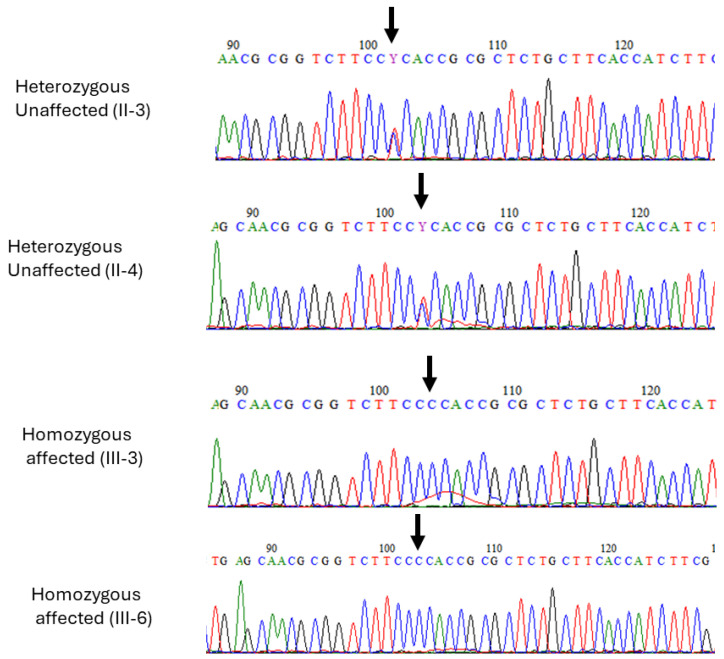
Sanger sequence analysis of missense variant in *SLC30A10* (c.266T>C) in the two affected individuals (III-3), (III-6) and heterozygous carriers (II-3) and mother (II-4). Black arrows in all electropherograms indicate the position of the identified variant.

Exome sequencing was performed on DNA samples obtained from both affected individuals (III-3) and (III-6). The sequencing generated high-quality reads with a coverage of over 100x. Through a comprehensive bioinformatics analysis of the exome data, a homozygous missense variant (c.266T>C; p.L89P) in *SLC30A10* was identified. This is in the first exon of the *SLC30A10* gene and results in the substitution of leucine with proline at codon number 89 of the protein (p.L89P). This variant was classified as deleterious and damaging by CADD and SIFT, respectively. Moreover, it is reported as pathogenic in ClinVar and disease-causing in HGMD (Table 2). To confirm the segregation of the variant, Sanger sequencing was conducted on DNA samples from all family members (Figure 3). Both affected individuals (III-3 and III-6) carried a homozygous mutant allele, while both parents and healthy siblings carried a heterozygous variant. This variant was, therefore, predicted to be the most likely pathogenic alteration associated with the observed clinical features in the patients.

### 3.4. In Silico Analysis and Protein Network Interaction

**Figure 4:**
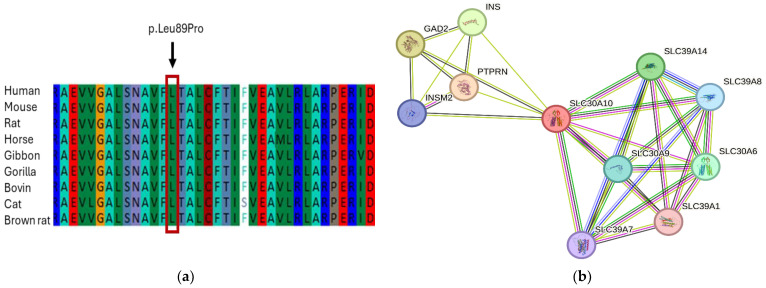
In silico analysis of SLC30A10. (**a**) SLC30A10 protein domain showing p. Leu89Pro is highly conserved in all vertebrate species from human to brown rat; (**b**) the protein network analysis of SLC30A10 was performed using the STRING software (Version 12). The PPI analysis showed strong interaction of SLC30A10 protein with other solute carrier family proteins as well as other vital proteins such as PTPRN, INSM2, INS, and GAD2.

A multiple sequence alignment was conducted to check the conservation of the (c.266T>C; p.L89P) variant. The alignment demonstrated that the leucine amino acid at this location is highly conserved across all vertebrate species from human, mouse, rat, horse, bovine, gorilla, cat to brown rat (Figure 4a). This finding suggests the significance of the leucine amino acid at position 89 in maintaining the structure and function of SLC30A10.

The protein network analysis was performed using the STRING software to understand the potential connections between SLC30A10 and other protein interacting partners. Our data showed a high-level association between SLC30A10 and other members of solute carrier family 30 and solute carrier family 39, which play important roles in the transportation and regulation of metals through the cellular membrane (Figure 4b). These proteins include SLC30A6, SLC30A9, SLC39A1, SLC39A7, SLC39A8, and SLC39A4. In addition, the network showed a strong association with other vital proteins such as protein tyrosine phosphatase receptor (PTPRN), INSM transcriptional repressor 2 (INSM2), insulin (INS), and glutamate decarboxylase 2 (GAD2).

## 4. Discussion

HMNDYT1 is one of the most underdiagnosed disorders. It is a rare condition that is inherited in families as an autosomal recessive disease [17]. The main neurological symptoms of HMNDYT1 in childhood include bradykinesia, dystonia, dysarthria, fine motor dysfunction, fine tremor, and, occasionally, spastic paraplegia [12,13,21]. Additionally, affected individuals exhibit non-neurological features such as polycythemia, elevated manganese levels in the blood, and hepatomegaly (enlarged liver) accompanied by varying degrees of hepatic fibrosis or cirrhosis [13]. In adulthood, patients with HMNDYT1 may also develop parkinsonism [21]. However, thus far, there is a lack of consensus regarding the clinical diagnostic criteria for this condition. 

In the present study, we reported a Saudi family with two children affected with segregated HMNDYT1 syndrome in an autosomal inherited recessive pattern. The patients exhibited symptoms of severe fine motor impairments, spastic paraparesis, postural instability, rigidity, bradykinesia, hypertonia of the limbs, and dystonia. Due to the heterogeneous characteristic of the syndrome and difficulty of exact diagnosis, we conducted exome sequencing for both patients and identified a homozygous missense variant (c.266T>C) in the *SLC30A10* gene. This mutation is in a highly conserved transmembrane region of the *SLC30A10* gene and has been previously described in three Arab sisters who exhibited syndromes of dystonia, liver cirrhosis, hypermanganesemia, and polycythemia [13]. Our current study supports the hypothesis of the involvement of the c.266T>C variant in the *SLC30A10* gene to cause this specific syndrome in an autosomal recessive manner. To our knowledge, this is the first report of specific c.266T>C mutation of *SLC30A10* from Saudi Arabia and broadens the mutation continuum of *SLC30A10* and phenotypical heterogeneity of the disease. 

In 2012, Tuschl et al. found that a yeast strain model carrying the missense variant (c.266T>C) or nonsense variant (c.585del) was unable to restore Mn resistance, in contrast to the wildtype strain [13]. Additionally, knockout mice of *Slc30a10* -/- showed high levels of Mn in the blood serum, hypothyroidism, a smaller size, and premature death compared to controls. Interestingly, the phenotype of the disease was more evident and appeared earlier in male mice than female mice [22]. This aligns with our patients, where the affected male developed the disease earlier, at the end of the first year, and the disease progressed rapidly compared to his sister, who developed the disease after 2 years and progressed slowly. However, a previous report by Tuschl et al. on the (c.266T>C) variant was on three affected Arab sisters who developed HMNDYT1 after the second year of life [13] and no male has been reported in this variant yet.

Manganese is an essential cation, and it has a significant impact on the homeostasis of various neurotransmitter systems. Excessive levels of Mn induce cellular toxicity through various mechanisms such as affecting DNA replication and transcription, triggering apoptotic cell death, ATP depletion, and interfering with mitochondrial oxidative function [12,23]. Our patient’s MRI report revealed neurodegeneration accompanied by the accumulation of iron/or manganese in the globus pallidus of the basal ganglia. However, exome sequencing identified a variant (c.266T>C) in *SLC30A10* which confirmed manganese intoxication was present in the globus pallidus. These findings are consistent with several previous reports showing similar results [12,13,15].

The amino acid alignment analysis showed a high conservation level of SLC30A10 at the p.(leu89Pro) region among humans, mice, rats, horses, bovines, gorillas, cats, and brown rats. An in vitro experiment observed that the Leu89Pro mutation completely disrupts the transportation of SLC30A10 to the cell membrane [24]. Additionally, a study suggested that polar or charged residues present in the transmembrane domains of SLC30A10 play a functional role in the transport of manganese [25]. 

It is important to emphasize that chelation therapy has demonstrated successful normalization of Mn blood levels in patients with HMNDYT1 and has led to significant clinical improvement in this potentially life-threatening condition [12,17]. However, early detection and diagnosis are crucial to intervene before the disease progresses and a patient’s health deteriorates. Whole exome sequencing (WES) is recommended for individuals presenting with neurological or hepatic symptoms, as well as those with polycythemia and hypermanganesemia. 

While filtering strategies and in silico prediction tools can help assess variant pathogenicity, extensive comprehension of pathological and functional consequences can be challenging. In this study, certain limitations may have impacted the confirmation of disease-causing effects for the identified variants. Further investigation utilizing functional studies, including in vitro and in vivo analysis of the variant in model organisms, is necessary to validate the pathogenic effects of the identified variant.

## 5. Conclusions

In conclusion, our study focused on the clinical and genetic analysis of a Saudi family with two affected individuals diagnosed with HMNDYT1. We successfully identified a recurrent pathogenic variant (c.266T>C) in the *SLC30A10* gene as a cause for this disease. Early detection and treatment of HMNDYT1 are crucial as the condition can lead to severe brain and liver damage. A previously performed brain MRI helped determine the accumulation of iron and/or manganese in the globus pallidus. Monitoring the levels of manganese (Mn) and iron is essential due to the chemical and structural similarities between these elements, as they compete for the same serum binding protein (transferrin) and membrane transporter protein (DMT1).

## Figures and Tables

**Table 1:** Clinical features and laboratory tests observed in both probands.

Clinical Features	III-3	III-4
Age of onset (years)	2	1
Age at examination (years)	11	2
Hepatomegaly	No	No
Liver cirrhosis	Yes	No
Dystonia	Yes	Yes
Hypertonia of the limbs	Yes	Yes
Fine motor impairment	Severe	Severe
Tremor	No	No
Bradykinesia	No	No
Rigidity	Yes	Yes
Postural instability	Yes	Yes
Spastic paraparesis	Yes	Yes
**Laboratory Test Results**		
Polycythemia	Yes	Yes
Serum manganese	High	High
Hyperbilirubinemia, unconjugated	Yes	Yes
Low iron	Low	Low
Total iron-binding capacity	High	High
**Brain MRI Findings**		
Hyperintensities in the basal ganglia	Yes	N/A
White matter lesions	Yes	N/A
Anterior pituitary lesions	No	N/A

Laboratory investigations of both patients showed elevated levels of Mn element in the serum, as well as high levels of unconjugated bilirubin and high total iron-binding capacity. Additionally, both patients exhibited decreased iron levels in their blood. The complete blood count (CBC) results indicated polycythemia in both patients.

**Table 2:** In silico analysis of *SLC30A10* gene.

Gene	Chr	Exon	Nucleotide Variant	Protein Variant	SNP No.	Zygosity	gnomAD Freq
*SLC30A10*	1	1	c.266T>C	p.L89P	rs281860284	Homo	0.0
**Prediction bioinformatics tools**							
**SIFT** **(v4.7)**	**CADD** **(v1.7)**	**VarSome**	**PolyPhen** **(v2)**	**PhyloP** **(V5.20)**	**ClinVar**	**HGMD** **variant class** **(v 2023.1)**	**ACMG** **classification**
D	De	P	PD	HC	P	DC	VUS

Abbreviation: D, Damaging; De, Deleterious; DC, Disease causing; HC, Highly Conserved; P, Pathogenic; PD, Probably Damaging.

## Data Availability

All data are available upon request.
